# *Lactiplantibacillus plantarum* GUANKE and Its Metabolite Phenyllactic Acid Reduce Intestinal *Klebsiella pneumoniae* Dysbacteriosis and Related Gut Inflammatory Injury

**DOI:** 10.3390/microorganisms14091921

**Published:** 2026-08-31

**Authors:** Jielan Mi, Xiao Zhang, Jing Liu, Kun Yue, Zhihan Yang, Yujia He, Yuanming Huang, Liqiong Song, Zhihong Ren, Jianguo Xu

**Affiliations:** 1 School of Medicine, Nankai University, Tianjin 300071, China; mijielan1007@163.com; 2 National Key Laboratory of Intelligent Tracking and Forecasting for Infectious Diseases, National Institute for Communicable Disease Control and Prevention, Chinese Center for Disease Control and Prevention, Beijing 102200, Chinalj2837901559@163.com (J.L.); kunyue95@163.com (K.Y.); xiaohanyzh@163.com (Z.Y.); ibukihe@163.com (Y.H.); huangyuanming@icdc.cn (Y.H.); songliqiong@icdc.cn (L.S.); 3 School of Public Health, Gansu University of Chinese Medicine, Lanzhou 730000, China; 4 Research Unit for Unknown Microbe, Chinese Academy of Medical Sciences & Peking Union Medical College, Beijing 100006, China

**Keywords:** *Klebsiella pneumoniae*, *Lactiplantibacillus plantarum* GUANKE, phenyllactic acid, intestinal KP burden, gut microbiota, influenza A virus

## Abstract

*Klebsiella pneumoniae* (KP) is an important opportunistic pathogen that can persist in the gastrointestinal tract and serve as a reservoir for subsequent infection. The increasing prevalence of multidrug-resistant KP highlights the need for non-antibiotic strategies to limit intestinal KP burden and associated host injury. *Lactiplantibacillus plantarum* GUANKE (*L. plantarum* GUANKE) is a candidate probiotic strain with reported effects on mucosal barrier protection and inflammatory regulation, but its role in intestinal KP challenge remains unclear. In this study, the effects of *L. plantarum* GUANKE and its metabolite phenyllactic acid (PLA) on KP growth, intestinal KP burden, and associated inflammatory injury were investigated using antibacterial assays in vitro, metabolomic analyses, an antibiotic-pretreated mouse model of intestinal KP challenge, and an influenza A virus (IAV)/KP intestinal co-exposure model. *L. plantarum* GUANKE and its culture-derived products inhibited KP growth in vitro. Metabolomic analysis identified PLA as a metabolite enriched in *L. plantarum* GUANKE fermentation supernatants, and exogenous PLA directly inhibited KP growth in a dose-dependent manner. In an antibiotic-pretreated mouse model of intestinal KP challenge, oral administration of *L. plantarum* GUANKE reduced intestinal KP burden and was associated with changes in gut microbiota composition, increased cecal PLA abundance, improved intestinal barrier-related parameters, and reduced inflammatory responses, while exogenous PLA partially reproduced these effects. IAV infection increased susceptibility to intestinal KP expansion, and oral *L. plantarum* GUANKE reduced KP burden in the IAV/KP intestinal co-exposure model. In mice challenged with multidrug-resistant KP strain NK04152, *L. plantarum* GUANKE and PLA reduce intestinal KP burden and associated tissue injury in mice, supporting their potential as candidate microbiota and metabolite strategies against intestinal KP challenge.

## 1. Introduction

*Klebsiella pneumoniae* (KP) is a major opportunistic pathogen associated with severe healthcare-associated infections, including pneumonia, bacteremia, urinary tract infection, and sepsis [1,2,3]. The increasing prevalence of multidrug-resistant and hypervirulent strains has further complicated the prevention and treatment of KP infections worldwide [4,5,6]. The gastrointestinal tract serves as an important reservoir for KP, where intestinal carriage may contribute to subsequent extraintestinal infection and transmission [7,8,9,10,11]. In healthy hosts, colonization resistance mediated by the gut microbiota and mucosal immune system limits KP expansion. Previous studies have demonstrated that commensal microorganisms can restrict KP establishment through microbiota-dependent and immune-mediated mechanisms, whereas disruption of these protective barriers increases susceptibility to KP expansion. Once this protective barrier is disrupted, KP may persist at high abundance, disseminate to other hosts or anatomical sites, and directly aggravate intestinal injury through the induction of epithelial and inflammatory responses [12]. Therefore, restoring intestinal colonization resistance and reducing KP burden may simultaneously limit intestinal damage, transmission, and the risk of subsequent invasive infection.

Intestinal colonization resistance can be disrupted by external perturbations. Broad-spectrum antibiotic exposure reduces microbial diversity and depletes protective commensals, thereby facilitating the expansion of drug-resistant opportunistic pathogens such as KP [12]. Respiratory viral infections may also influence intestinal microbial and immune homeostasis. In particular, influenza A virus (IAV) infection has been reported to alter gut microbiota composition and mucosal immune responses, which may affect susceptibility to secondary bacterial complications [13,14,15,16,17]. Although influenza-associated bacterial co-infection is closely associated with increased disease severity and mortality, most studies have focused on secondary respiratory infections, whereas the possibility that IAV-induced intestinal dysbiosis and mucosal immune disturbance may increase susceptibility to enteric KP expansion after oral exposure remains insufficiently explored [18,19]. This gap provides a rationale for establishing an IAV-associated KP intestinal exposure model to examine whether respiratory viral infection creates a permissive intestinal context for KP expansion and whether such expansion can be attenuated by microbiota-based intervention.

Increasing evidence has shown that probiotics and their derived metabolites play important roles in maintaining intestinal homeostasis, suppressing pathogen infection, and alleviating inflammatory injury [20]. Therefore, microbiota- and metabolite-based interventions may provide promising non-antibiotic strategies for intestinal KP colonization and associated injury. *Lactiplantibacillus plantarum* GUANKE (*L. plantarum* GUANKE) is a functional strain isolated from the feces of healthy volunteers. Previous safety evaluations, together with studies in multiple animal models and human studies, provide support for its safety and translational potential [21,22,23,24]. Moreover, its reported effects on mucosal barrier integrity, antiviral immunity, and inflammatory regulation provide a rationale for evaluating *L. plantarum* GUANKE against intestinal KP colonization [22].

Lactobacilli produce a variety of organic acids that contribute to their antimicrobial activity by acidifying the local environment and directly interfering with pathogen growth. Among these metabolites, phenyllactic acid (PLA) is an aromatic organic acid produced by diverse lactic acid bacteria and related genera, including *Lactobacillus*, *Lactiplantibacillus*, *Bifidobacterium*, *Companilactobacillus*, and *Lactococcus* species [25,26,27,28,29,30,31,32,33,34]. PLA has been reported to inhibit the growth of multiple bacterial and fungal pathogens and to interfere with biofilm formation [35,36]. Beyond its direct antimicrobial activity, PLA has also been associated with anti-inflammatory regulation, intestinal metabolic homeostasis, and antiviral effects [37,38,39,40]. Notably, PLA has been shown to antagonize KP growth in vitro [41]. The present study extends this previous finding by evaluating whether PLA producing *L. plantarum* GUANKE can antagonize intestinal KP expansion and associated tissue injury in vivo, and by further examining its effect under IAV/KP co exposure conditions.

Therefore, this study investigated whether *L. plantarum* GUANKE and PLA could reduce intestinal KP burden and associated tissue injury using in vitro assays and mouse models. This study further examined whether IAV infection altered susceptibility to intestinal KP expansion and whether *L. plantarum* GUANKE provided protection under this condition.

## 2. Materials and Methods

### 2.1. Bacteria, Cells and Viruses

*L. plantarum* GUANKE (CGMCC No. 21720) was deposited at the China General Microbiological Culture Collection Center. *L. paracasei* SUB12, *L. sakei* QZ1, and *L. pentosus* P2020 were preserved in our laboratory. All lactobacilli were stored at −80 °C in MRS broth containing 30% glycerol, revived on de Man Rogosa Sharpe (MRS) agar plates, and statically cultured at 37 °C under 5% CO_2_ for 24 h before use.

The multidrug-resistant KP strain NK04152 (NCBI BioSample accession: SAMN62590419) was kindly provided by Dr. Jie Feng from the Institute of Microbiology, Chinese Academy of Sciences. NK04152 is a clinical isolate belonging to sequence type 11 (ST11) and capsule serotype K64. KP was stored at −80 °C in brain heart infusion (BHI) broth containing 30% glycerol, revived on BHI agar plates, and statically cultured at 37 °C under 5% CO_2_ for 24 h. Before use, KP colonies were collected from BHI agar plates, suspended in PBS, and adjusted to a McFarland turbidity of 3.0, corresponding to approximately 1 × 10^9^ CFU/mL.

Madin–Darby canine kidney (MDCK) cells were preserved in our laboratory and cultured in Dulbecco’s modified Eagle’s medium (DMEM, Gibco, Grand Island, NY, USA) supplemented with 10% fetal bovine serum (FBS, Gibco, Grand Island, NY, USA) and 1% penicillin streptomycin (Gibco, Grand Island, NY, USA). Cells were maintained at 37 °C in a humidified incubator with 5% CO_2_ and passaged at approximately 85% confluence using trypsin digestion.

The mouse adapted influenza A/Puerto Rico/8/34 (PR8, H1N1) virus used in this study was obtained from the Chinese National Influenza Center (CNIC) and stored at −80 °C until use.

### 2.2. Primers

All primers were designed based on target gene sequences retrieved from the NCBI database and synthesized by Rui Biotech Co., Ltd. (Beijing, China). The primer sequences are listed in Table 1.

### 2.3. Preparation of Bacterial Fermentation Supernatant

Bacterial strains were revived from glycerol stocks stored at −80 °C and streaked onto MRS agar plates. After static incubation at 37 °C for 24 h in a temperature-controlled incubator (Thermo Fisher Scientific, Waltham, MA, USA), a single colony from a freshly grown MRS agar plate was selected and inoculated into 5 mL of MRS broth. The culture was then incubated statically at 37 °C under 5% CO_2_ for 24 h. Subsequently, the bacterial culture was centrifuged at 4000 rpm for 10 min using a high-speed refrigerated centrifuge (Eppendorf, Hamburg, Germany), and the supernatant was collected and filtered through a sterile 0.45 μm membrane filter (Merck, Darmstadt, Germany) to obtain cell-free fermentation supernatant (CFS) for subsequent experiments.

### 2.4. Antibacterial Activity Assay In Vitro

The inhibitory effects of *L. plantarum* GUANKE bacterial cells, bacterial fermentation supernatants, and PLA on KP were evaluated by plate colony counting. For the co-culture assay, KP was mixed with *L. plantarum* GUANKE in MRS broth at a 1:1 ratio, with 1 × 10^9^ CFU of each bacterium, and incubated statically at 37 °C for 1, 6, or 24 h. To assess ratio-dependent inhibition, KP was co-cultured with *L. plantarum* GUANKE at different GUANKE:KP ratios, while the KP inoculum was kept constant at 1 × 10^9^ CFU. KP incubated in MRS broth alone under the same conditions was used to exclude the interference of MRS medium on KP growth.

For the cell-free supernatant assay, 1 × 10^9^ CFU of KP was incubated with 1 mL of *L. plantarum* GUANKE fermentation supernatant at 37 °C for 1 h. Heat-inactivated supernatant was prepared by heating the supernatant at 95 °C for 10 min. To evaluate the contribution of acidity, MRS medium and *L. plantarum* GUANKE fermentation supernatant were adjusted to the indicated pH values using 2 M HCl or NaOH before incubation with KP. In parallel, KP was incubated with fermentation supernatants from *L. paracasei* SUB12, *L. sakei* QZ1, and *L. pentosus* P2020, or with different concentrations of PLA, under the same conditions. After incubation, viable KP cells were enumerated on HiCrome™ *Klebsiella* Selective Agar Base (Himedia Laboratories, Thane, India). All assays were performed in three independent biological experiments, with each condition assessed in technical triplicate.

### 2.5. Mice

Female SPF-grade C57BL/6J mice, 4–6 weeks old and weighing 12–14 g, were purchased from Beijing Vital River Laboratory Animal Technology Co., Ltd. (Beijing, China). and housed under specific pathogen-free conditions at the Animal Center of the Chinese Center for Disease Control and Prevention. Mice were maintained with autoclaved corncob bedding under controlled conditions of 22 ± 2 °C, 50 ± 10% relative humidity, and a 12-h light/dark cycle, with sterilized chow and water provided ad libitum.

Animals were monitored at least once daily. Predefined humane endpoints included more than 20% loss of initial body weight, severe lethargy, or inability to access food or water. No animals reached these endpoints, and no animals, samples, or data points were excluded. All procedures were approved by the Laboratory Animal Welfare Ethics Committee of the Chinese Center for Disease Control and Prevention (approval number: 2024-043; approval date: 1 August 2024).

### 2.6. Mouse Models

Mice were randomly assigned to experimental groups, with six mice per group and three mice housed per cage. The sample size was selected based on preliminary experimental observations, similar published animal studies, and ethical principles aimed at minimizing animal use.

For the KP colonization model, mice were pretreated with a broad-spectrum antibiotic cocktail by oral gavage for 5 consecutive days, consisting of ampicillin (200 mg/kg, Sigma, St. Louis, MO, USA), vancomycin (100 mg/kg, Sigma, St. Louis, MO, USA), neomycin sulfate (200 mg/kg, Sigma, St. Louis, MO, USA), and metronidazole (200 mg/kg, Sigma, St. Louis, MO, USA). The antibiotic cocktail was administered in 100 μL per mouse. Antibiotic pretreatment was used to disrupt colonization resistance and establish a reproducible, permissive intestinal environment for KP colonization, as described in previous studies [12,42]. All groups in this model, including the antibiotic-treated non-KP control group (Blank group), received the same antibiotic pretreatment regimen. Mice in all KP-challenged groups were orally administered 200 μL of KP suspension containing 5 × 10^8^ CFU. *L. plantarum* GUANKE was administered after KP challenge to evaluate its therapeutic effect on intestinal KP burden. At 6 h after KP challenge, daily oral administration was initiated and continued for 11 consecutive days. Mice in the KP + GK group received 200 μL of PBS containing 5 × 10^9^ CFU of *L. plantarum* GUANKE per mouse, while mice in the KP + PLA group received 200 μL of PLA solution at 40 mg/kg. Mice in the other groups received an equal volume of PBS according to the same schedule. Fecal samples were collected on days 1, 3, 5, 7, 9, and 11 after KP challenge, and the number of KP colonies in feces was determined using HiCrome™ *Klebsiella* Selective Agar Base (Himedia Laboratories, Thane, India). On day 11, the mice were euthanized under anesthesia by cervical dislocation. Colon tissues were collected for subsequent experiments and colon length was measured, while cecal contents were harvested for 16S rRNA sequencing and untargeted metabolomic analysis.

For the IAV/KP intestinal co-exposure model, mice were first anesthetized by intraperitoneal injection of pentobarbital sodium at 40 mg/kg and then infected intranasally with IAV at a sublethal dose of 240 PFU per mouse, as established in previously published protocol [22,43]. This dose was selected to induce measurable pulmonary inflammation without causing mortality during the observation period. On day 3 or day 5 after IAV infection, KP was administered by oral gavage at 5 × 10^9^ CFU per mouse in a total volume of 200 μL. At 6 h after KP challenge, mice in the IAV + KP + GK group were orally administered 200 μL of PBS containing 5 × 10^9^ CFU of *L. plantarum* GUANKE per mouse, whereas mice in the KP group and the IAV + KP group received an equal volume of PBS. At 24 h after KP challenge, fecal samples were collected for KP enumeration. The mice were euthanized by cervical dislocation while under anesthesia, and cecal contents were collected for 16S rRNA sequencing. Lungs were harvested and weighed for the calculation of the lung index according to Formula (1). The lung tissues were subsequently snap-frozen in liquid nitrogen and transferred to a −80 °C freezer for further analysis.

Lung index = Lung weight/Body weight × 100%(1)

### 2.7. Real-Time Quantitative PCR (RT-qPCR)

Total RNA was isolated from colon tissues and MLNs using RNAiso Plus (Takara, Dalian, China), and cDNA was synthesized with HiScript IV All-in-One Ultra RT SuperMix (Vazyme, Nanjing, China) for qPCR. Quantitative PCR was carried out using SYBR qPCR Mix (Vazyme, Nanjing, China) under the following cycling conditions: an initial step at 95 °C for 30 s, followed by 40 amplification cycles at 95 °C for 10 s, 58 °C for 20 s, and 72 °C for 30 s, and then a melting curve procedure consisting of 95 °C for 15 s, 60 °C for 1 min, and 95 °C for 15 s. Relative transcript levels were determined using the 2^−ΔΔCT^ method and normalized to β-actin. Colon and MLN samples from six individual mice per group were analyzed, with each mouse considered an independent biological replicate. Each qPCR reaction was performed in technical triplicate.

### 2.8. Western Blotting

Representative immunoblots and densitometric analyses were generated using colon tissues from three individual mice per group as independent biological replicates. Colon tissues were homogenized using a tissue grinder, and total protein was extracted with RIPA buffer (Beyotime, Shanghai, China) supplemented with protease inhibitors. Protein concentrations were determined using a BCA Protein Assay Kit (LABLEAD, Beijing, China). Equal amounts of protein were separated by SDS-PAGE and then transferred onto polyvinylidene fluoride (PVDF) membranes (Millipore, Burlington, MA, USA). The membranes were blocked with 5% nonfat milk (BD Diagnostics, Sparks, MD, USA) for 2 h and incubated overnight at 4 °C with the indicated primary antibodies (Occludin, Cell Signaling Technology, Danvers, MA, USA; Claudin-1, Abcam, Cambridge, UK). After thorough washing with TBST, the membranes were incubated with HRP-conjugated secondary antibodies (Epizyme Biotech, Shanghai, China) at room temperature for 45 min. Following additional washing with TBST, protein bands were visualized using ECL reagent (LABLEAD, Beijing, China) with a fully automated multifunctional imaging system (Tanon CHEMI DOG ULTRA, Shanghai Tanon Life Science Co., Ltd., Shanghai, China). Band intensities were quantified using ImageJ (version 1.53e) software, and target protein expression was normalized to β-actin.

### 2.9. ELISA

Lung tissues (100 mg) were homogenized in 1 mL of ice-cold PBS and centrifuged at 3000 rpm for 10 min at 4 °C. The supernatants were collected, and the concentrations of IL-1β and TNF-α were measured using mouse-specific ELISA kits (Invitrogen, Carlsbad, CA, USA) according to the manufacturer’s instructions. Lung tissue samples from six individual mice in each group were analyzed, with each mouse considered an independent biological replicate. Each sample was assayed in technical triplicate.

### 2.10. Determination of Viral Titers

Viral titers in lung tissues were determined using the 50% tissue culture infectious dose (TCID_50_) assay. Briefly, lung tissues were homogenized in 1 mL of sterile PBS, and the homogenates were centrifuged to remove tissue debris. The resulting supernatants were serially diluted and inoculated onto confluent MDCK cell monolayers in 96-well plates, with four replicate wells for each dilution. Following incubation, the cells were examined for cytopathic effects (CPEs), and the numbers of CPE-positive and CPE-negative wells at each dilution were recorded. The TCID_50_ values were calculated using the Reed–Muench method and expressed as log_10_ TCID_50_/100 μL.

### 2.11. Metabolomic Analysis

Untargeted metabolomic profiling of bacterial fermentation supernatants and cecal contents was performed by Shanghai Majorbio Bio-pharm Technology Co., Ltd. (Shanghai, China). Samples were extracted using the corresponding solvent systems and analyzed using a Vanquish Horizon UHPLC system coupled to an Orbitrap Exploris 240 mass spectrometer (Thermo Scientific, Waltham, MA, USA) in positive- and negative-ion modes. Pooled quality-control samples were prepared by mixing equal aliquots of all sample extracts and were inserted after every 5–15 analytical samples to monitor analytical stability. Raw data were processed using Progenesis QI version 3.0 for peak detection, alignment, integration, and metabolite annotation against HMDB, METLIN, and an in-house database. Features with more than 20% missing values in each group were removed, and the remaining missing values were replaced with the minimum detected value. Peak intensities were normalized by total-sum normalization, variables with QC RSD values greater than 30% were excluded, and the resulting data were log10-transformed. PCA and OPLS-DA were performed using the ropls package version 1.6.2 in R, and model reliability was evaluated by cross-validation and 200 permutation tests. For differential metabolite analysis, unpaired two-tailed Student’s *t*-test was used for pairwise comparisons, and the resulting *p*-values were adjusted for multiple testing using the Benjamini–Hochberg false discovery rate method. OPLS-DA-derived VIP values were used to evaluate the contribution of each metabolite to group separation. Differentially abundant metabolites were defined as those with FDR < 0.05 and OPLS-DA-derived VIP > 1. Metabolite abundance was expressed as normalized LC–MS peak area rather than absolute concentration.

Targeted quantification of organic acid in bacterial culture supernatants was conducted by Shanghai Sangon Biotech Co., Ltd. (Shanghai, China). Briefly, 50 μL of each sample was mixed with 250 μL of 20% acetonitrile–methanol extraction solution, vortexed for 3 min, and centrifuged at 12,000 rpm for 10 min at 4 °C. A 250 μL aliquot of the supernatant was transferred to a new tube, incubated at −20 °C for 30 min, and centrifuged again under the same conditions. Subsequently, 180 μL of the final supernatant was subjected to LC–MS/MS analysis using a Waters ACQUITY H-Class UPLC system coupled to a SCIEX QTRAP 6500+ mass spectrometer equipped with an ACQUITY UPLC BEH Amide column. PLA was identified by comparison with an authentic standard based on its retention time and characteristic MRM transitions and was quantified using an external standard curve. Mixed QC samples were inserted approximately once every 10 analytical samples to evaluate analytical stability and reproducibility.

### 2.12. 16S rRNA Sequencing of Intestinal Microbiota

Fresh mouse cecal contents were collected, snap-frozen in liquid nitrogen, and stored at −80 °C until use. Six mice per group were analyzed, with each mouse considered an independent biological replicate. Amplification and sequencing of the V3–V4 region of the bacterial 16S rRNA gene were performed by Shanghai Majorbio Bio-pharm Technology Co., Ltd. (Shanghai, China). The V3–V4 region of the bacterial 16S rRNA gene was amplified using primers 338F (5′-ACTCCTACGGGAGGCAGCAG-3′) and 806R (5′-GGACTACHVGGGTWTCTAAT-3′). PCR amplification was performed for each sample, and the products were pooled, purified, and subjected to paired-end sequencing on an Illumina NextSeq 2000 platform. Raw reads were quality-filtered using fastp version 0.23.4, merged using FLASH version 1.2.11, and denoised using the DADA2 plugin in QIIME2 to generate amplicon sequence variants. Taxonomic assignment was performed against the SILVA 138.2 database using the classify-sklearn naïve Bayes classifier with a 70% confidence threshold. Chloroplast and mitochondrial sequences were removed, and the ASV table was rarefied to an equal sequencing depth before downstream analysis. Alpha diversity was evaluated using the Chao and Shannon indices. Beta diversity was assessed by principal coordinate analysis based on Bray–Curtis dissimilarities. Overall differences among groups were tested using PERMANOVA implemented with the Adonis function with 999 permutations. Pairwise PERMANOVA was further performed, and the resulting *p*-values were adjusted using the Benjamini–Hochberg method. Differences in alpha-diversity indices and taxonomic abundances were assessed using the Kruskal–Wallis or Wilcoxon rank-sum test.

### 2.13. Statistical Analysis

Data were analyzed using GraphPad Prism v8.0. Comparisons between two groups were performed using a two-tailed unpaired Student’s *t*-test, while comparisons among multiple groups were performed using one-way ANOVA followed by Tukey’s multiple-comparisons test. Longitudinal fecal KP burdens were analyzed using two-way repeated-measures ANOVA after log10 transformation. Data are presented as mean ± SD, and each mouse was considered an independent biological replicate. A value of *p* < 0.05 was considered statistically significant (*, *p* < 0.05; **, *p* < 0.01; ***, *p* < 0.001; ****, *p* < 0.0001).

## 3. Results

### 3.1. L. plantarum GUANKE and PLA Exhibit Inhibitory Activity Against KP Growth In Vitro

**Figure 1:**
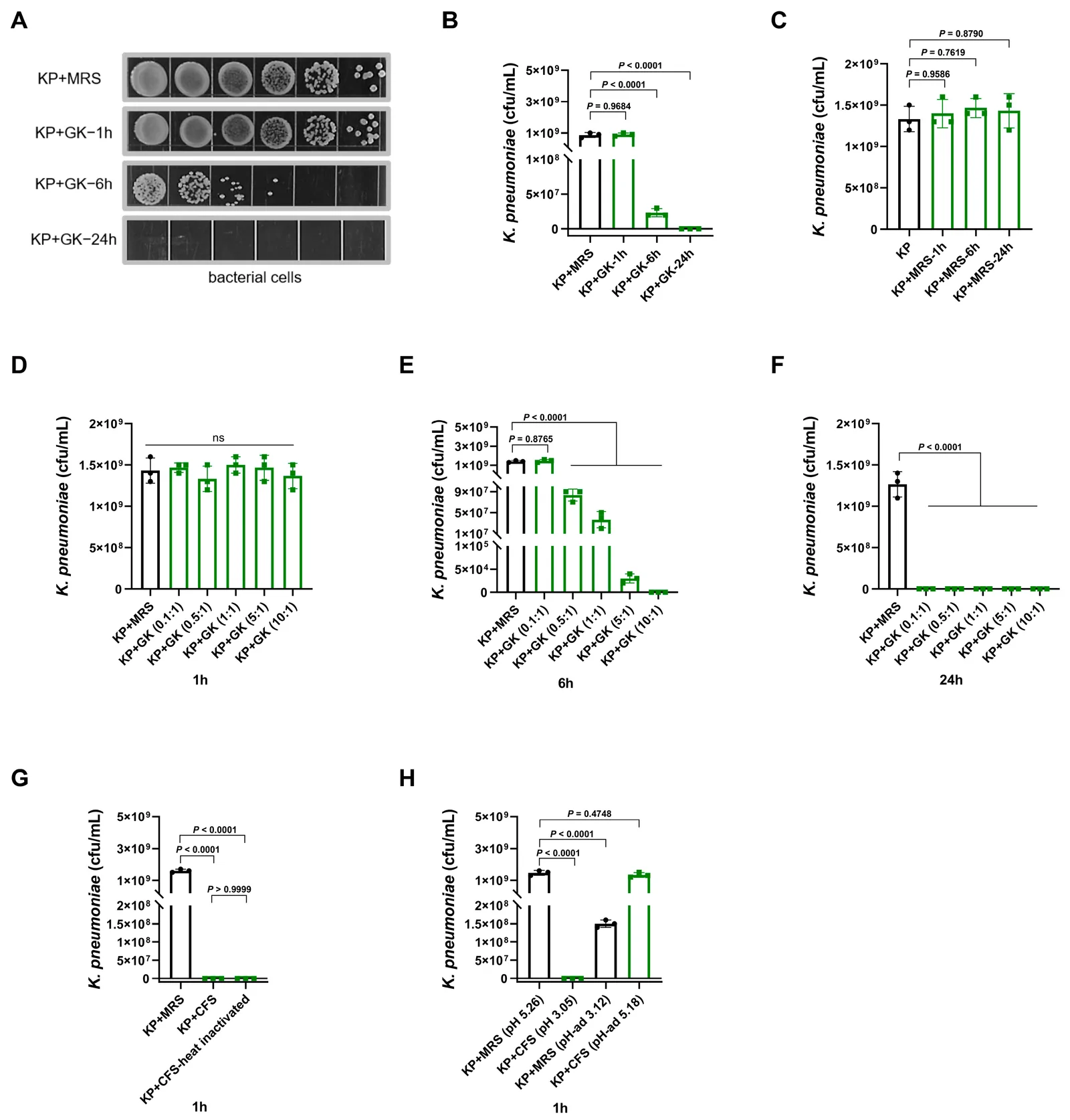
*L. plantarum* GUANKE inhibits KP growth through time-dependent, ratio-dependent, and acidity-associated effects in vitro. Representative plate images showing KP growth after co-culture with *L. plantarum* GUANKE bacterial cells and KP at a 1:1 ratio for 1, 6, and 24 h (**A**), and corresponding viable KP counts (**B**) (*n* = 3). Viable KP counts after incubation with MRS medium for 1, 6, and 24 h (**C**) (*n* = 3). Viable KP counts after co-culture with different ratios of *L. plantarum* GUANKE and KP for 1 h (**D**), 6 h (**E**), and 24 h (**F**) (*n* = 3). Viable KP counts after treatment with *L. plantarum* GUANKE cell-free fermentation supernatant or heat-inactivated supernatant for 1 h (**G**) (*n* = 3). Viable KP counts after exposure to untreated MRS medium, original *L. plantarum* GUANKE cell-free supernatant, acidified MRS medium, or pH-adjusted *L. plantarum* GUANKE cell-free supernatant for 1 h (**H**) (*n* = 3). Data are presented as means ± SD. Statistical significance was set at *p* < 0.05; ns, not significant.

To investigate the antibacterial activity of *L. plantarum* GUANKE against KP, bacterial co-culture assays were first performed using *L. plantarum* GUANKE and KP at a 1:1 ratio. Co-culture with *L. plantarum* GUANKE had little effect on KP growth at 1 h, but markedly reduced viable KP counts at 6 h and almost completely inhibited KP growth at 24 h (Figure 1A,B). In contrast, incubation with MRS medium alone for the same durations did not significantly affect KP growth, thereby excluding the possibility that MRS medium itself interfered with KP growth (Figure 1C). The effect of different *L. plantarum* GUANKE-to-KP ratios was then evaluated. At 1 h, increasing the proportion of *L. plantarum* GUANKE did not significantly reduce KP counts (Figure 1D). However, at 6 h, KP growth was progressively suppressed as the proportion of *L. plantarum* GUANKE increased, and a stronger inhibitory effect was observed at higher ratios (Figure 1E). At 24 h, all tested ratios of *L. plantarum* GUANKE markedly inhibited KP growth (Figure 1F). These results indicate that *L. plantarum* GUANKE inhibits KP growth in a time- and ratio-dependent manner, suggesting that extracellular factors produced during *L. plantarum* GUANKE growth may contribute to KP inhibition.

Because the inhibitory effect became more evident after prolonged co-culture, the contribution of cell-free fermentation supernatant from *L. plantarum* GUANKE was further evaluated. Cell-free fermentation supernatant from *L. plantarum* GUANKE strongly reduced KP counts after 1 h of exposure, and heat inactivation did not abolish this inhibitory activity (Figure 1G). To further assess the contribution of acidity, pH-adjusted controls were included. Compared with untreated MRS medium, the original acidic *L. plantarum* GUANKE supernatant markedly inhibited KP growth. Acidified MRS medium also reduced KP counts, whereas pH-adjusted *L. plantarum* GUANKE supernatant showed substantially weakened antibacterial activity (Figure 1H). These findings suggest that acidity contributes importantly to the inhibitory activity of *L. plantarum* GUANKE fermentation supernatant. Untargeted metabolomic analysis of *L. plantarum* GUANKE fermentation supernatant further revealed a distinct metabolic profile compared with MRS medium (Figure S1).

**Figure 2:**
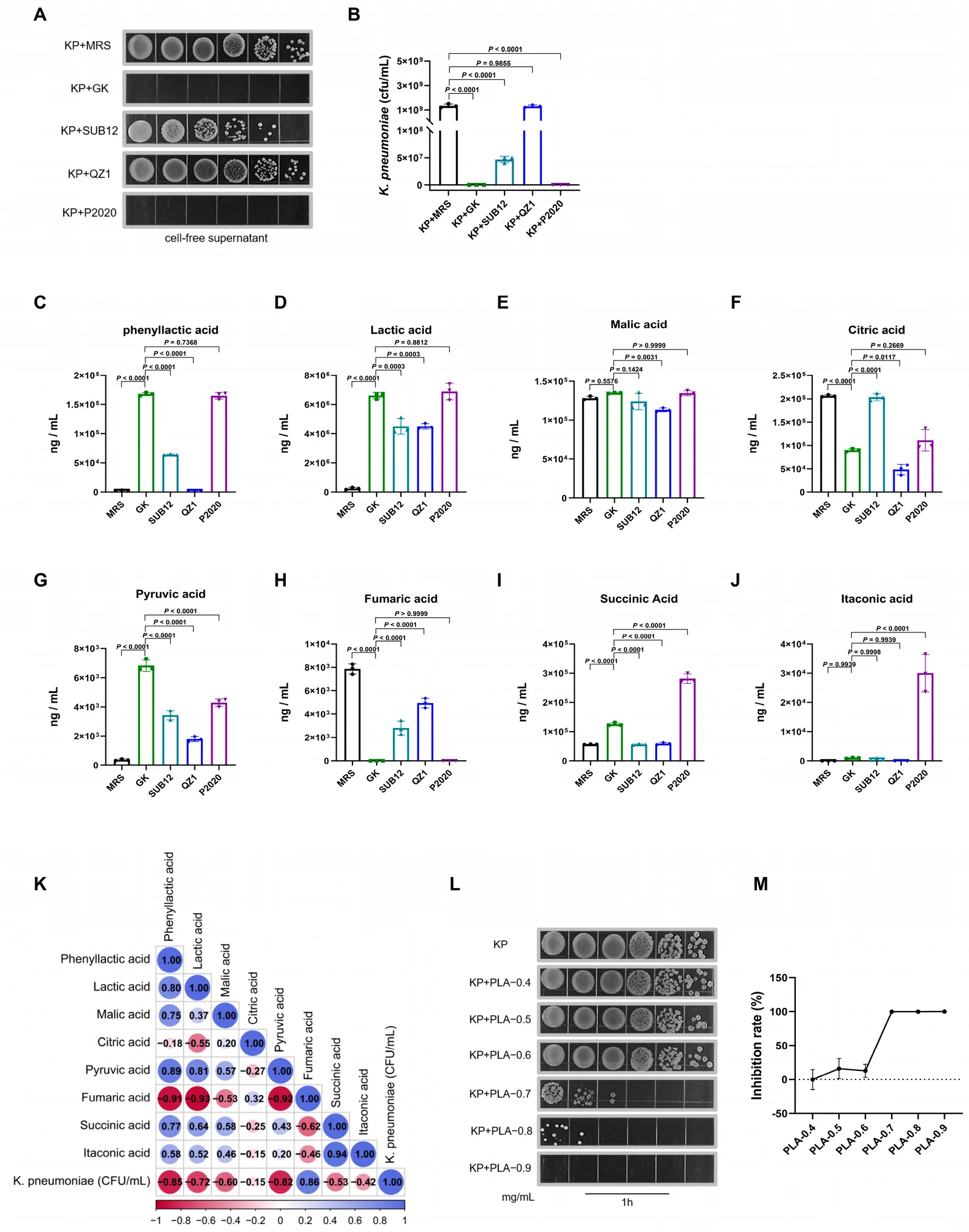
PLA is enriched in *L. plantarum* GUANKE fermentation supernatant and directly inhibits KP growth in vitro. Representative plate images showing KP growth after treatment with cell-free fermentation supernatants from *L. plantarum* GUANKE, *L. paracasei* SUB12, *L. sakei* QZ1, and *L. pentosus* P2020 (**A**), and corresponding viable KP counts (**B**) (*n* = 3). Concentrations of phenyllactic acid (**C**), lactic acid (**D**), malic acid (**E**), citric acid (**F**), pyruvic acid (**G**), fumaric acid (**H**), succinic acid (**I**), and itaconic acid (**J**) in MRS medium and bacterial fermentation supernatants (*n* = 3). Pearson correlation matrix showing associations among organic acid concentrations and viable KP counts across fermentation supernatant groups (**K**). Representative plate images showing KP growth after treatment with increasing concentrations of PLA (**L**), and corresponding inhibition rates (**M**) (*n* = 3). Data are presented as means ± SD. Statistical significance was set at *p* < 0.05.

The antibacterial activities of cell-free fermentation supernatants from different lactobacilli were next compared. Among the tested strains, supernatants from *L. plantarum* GUANKE and *L. pentosus* P2020 showed the strongest inhibition of KP growth, whereas *L. paracasei* SUB12 showed partial inhibition and *L. sakei* QZ1 showed little inhibitory effect under the same conditions (Figure 2A,B). Targeted quantification of representative organic acids showed that PLA was markedly enriched in the fermentation supernatants of *L. plantarum* GUANKE and *L. pentosus* P2020 compared with MRS medium and the other strains (Figure 2C). Other organic acids, including lactic acid, malic acid, citric acid, pyruvic acid, fumaric acid, succinic acid, and itaconic acid, also differed among the fermentation supernatants (Figure 2D–J). Correlation analysis further showed that, among the detected organic acids, PLA exhibited the strongest negative association with viable KP counts, supporting a potential contribution of PLA to the anti-KP activity of the fermentation supernatants (Figure 2K). Finally, exogenous PLA inhibited KP growth in a dose-dependent manner, with near-complete inhibition observed at concentrations of 0.7 mg/mL or higher (Figure 2L,M). Together, these findings indicate that *L. plantarum* GUANKE-derived products inhibit KP growth in vitro and support PLA as one candidate metabolite contributing to this inhibitory effect.

### 3.2. L. plantarum GUANKE and PLA Reduce Intestinal KP Burden In Vivo

**Figure 3:**
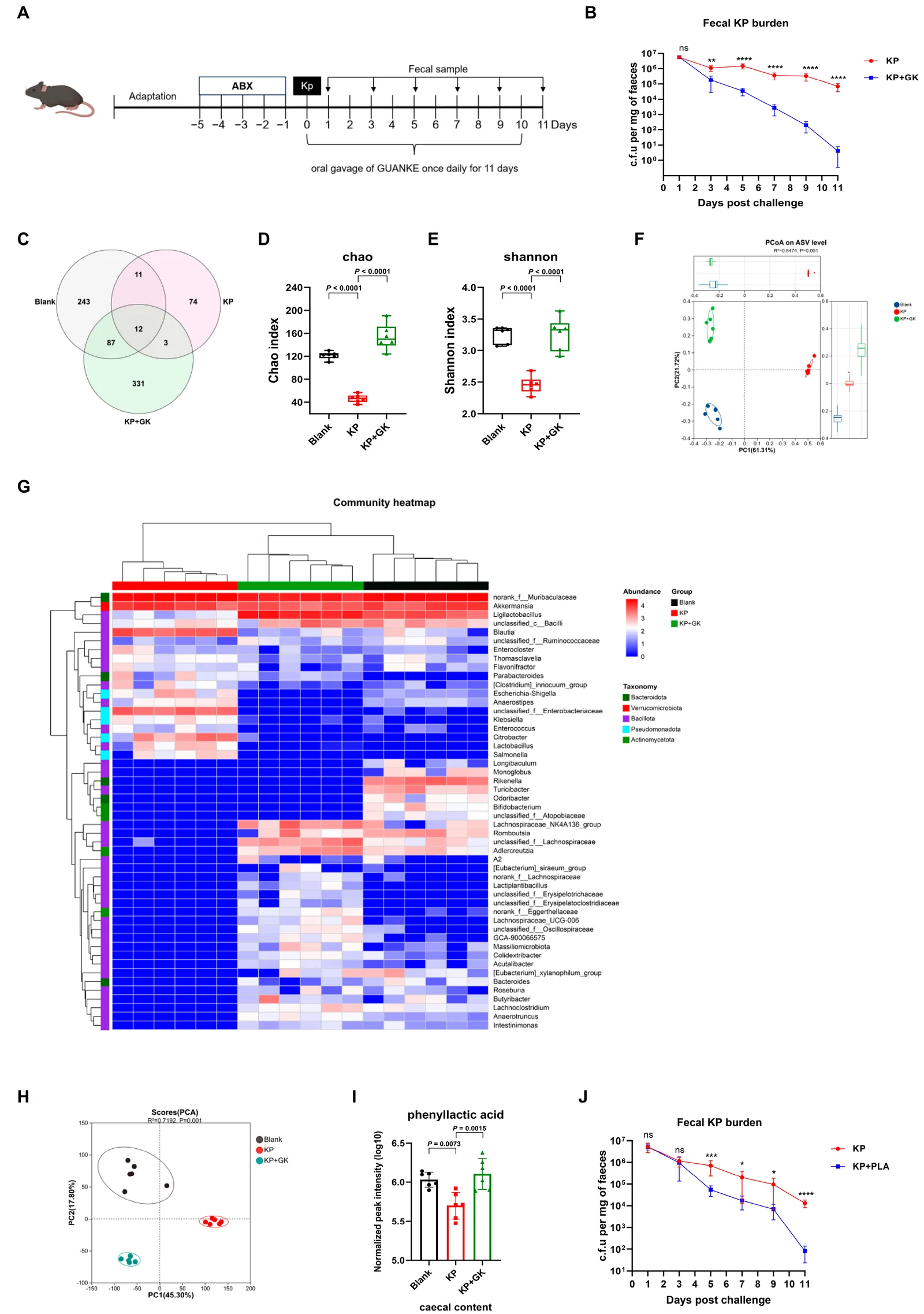
Oral administration of *L. plantarum* GUANKE reduces intestinal KP burden, is associated with changes in the gut microbiota and increased normalized cecal PLA peak intensity in vivo. Experimental design (**A**). Fecal KP burden over time in the KP and KP + GK groups (**B**). Venn diagram of shared and unique ASVs (**C**). Chao index (**D**) and Shannon index (**E**) of ASVs. PCoA showing differences in gut microbial community structure among the three groups (**F**). Community heatmap showing differences in gut microbial composition among the three groups (**G**). PCA of cecal metabolomic profiles (**H**). Normalized PLA peak intensity (log10) in cecal contents (**I**). Fecal KP burden over time in the KP and KP + PLA groups (**J**). Data are presented as means ± SD (*n* = 6 mice per group). Statistical significance was set at * *p* < 0.05, ** *p* < 0.01, *** *p* < 0.001, **** *p* < 0.0001, and ns, not significant.

To evaluate the effect of *L. plantarum* GUANKE on intestinal burden in vivo, mice were pretreated with antibiotics, challenged with KP, and then orally gavaged with *L. plantarum* GUANKE 6 h after KP challenge (Figure 3A). Two-way repeated-measures ANOVA revealed significant effects of treatment group, time, and their interaction on fecal KP burdens over time. Holm–Šídák-adjusted comparisons showed that *L. plantarum* GUANKE significantly reduced fecal KP burdens from day 3 onward (Figure 3B). 16S rRNA gene sequencing further showed that KP challenge disrupted gut microbial richness, diversity, and community structure, as evidenced by reduced Chao and Shannon indices and distinct clustering patterns, whereas *L. plantarum* GUANKE treatment was associated with higher diversity indices and changes in microbial community structure relative to the KP group (Figure 3C–G). PERMANOVA confirmed significant overall differences in microbial community composition among the three groups (*R*^2^ = 0.8474, *p* = 0.001), including between the KP and *L. plantarum* GUANKE groups (adjusted *p* = 0.006). In parallel, metabolomic analysis of cecal contents revealed clear group separation and increased normalized PLA peak intensity in *L. plantarum* GUANKE-treated mice relative to KP-challenged mice (Figure 3H,I). Similarly, two-way repeated-measures ANOVA showed significant effects of treatment group, time, and their interaction in the PLA experiment. Holm–Šídák-adjusted comparisons showed that PLA significantly reduced fecal KP burdens from day 5 onward (Figure 3J). In addition, KP bacterial translocation was also assessed in this model. KP was not detected in the heart, liver, spleen, lung, kidney, or whole blood by selective culture, indicating that KP challenge in this antibiotic pretreated model did not result in detectable bacterial translocation. Together, these findings indicate that *L. plantarum* GUANKE suppresses intestinal KP burden in vivo, is accompanied by changes in gut microbial community structure, and increases intestinal PLA levels, while the protective effects of exogenous PLA support a contributory role for PLA.

### 3.3. L. plantarum GUANKE and PLA Are Associated with Reduced KP-Associated Intestinal Barrier Damage and Inflammation

**Figure 4:**
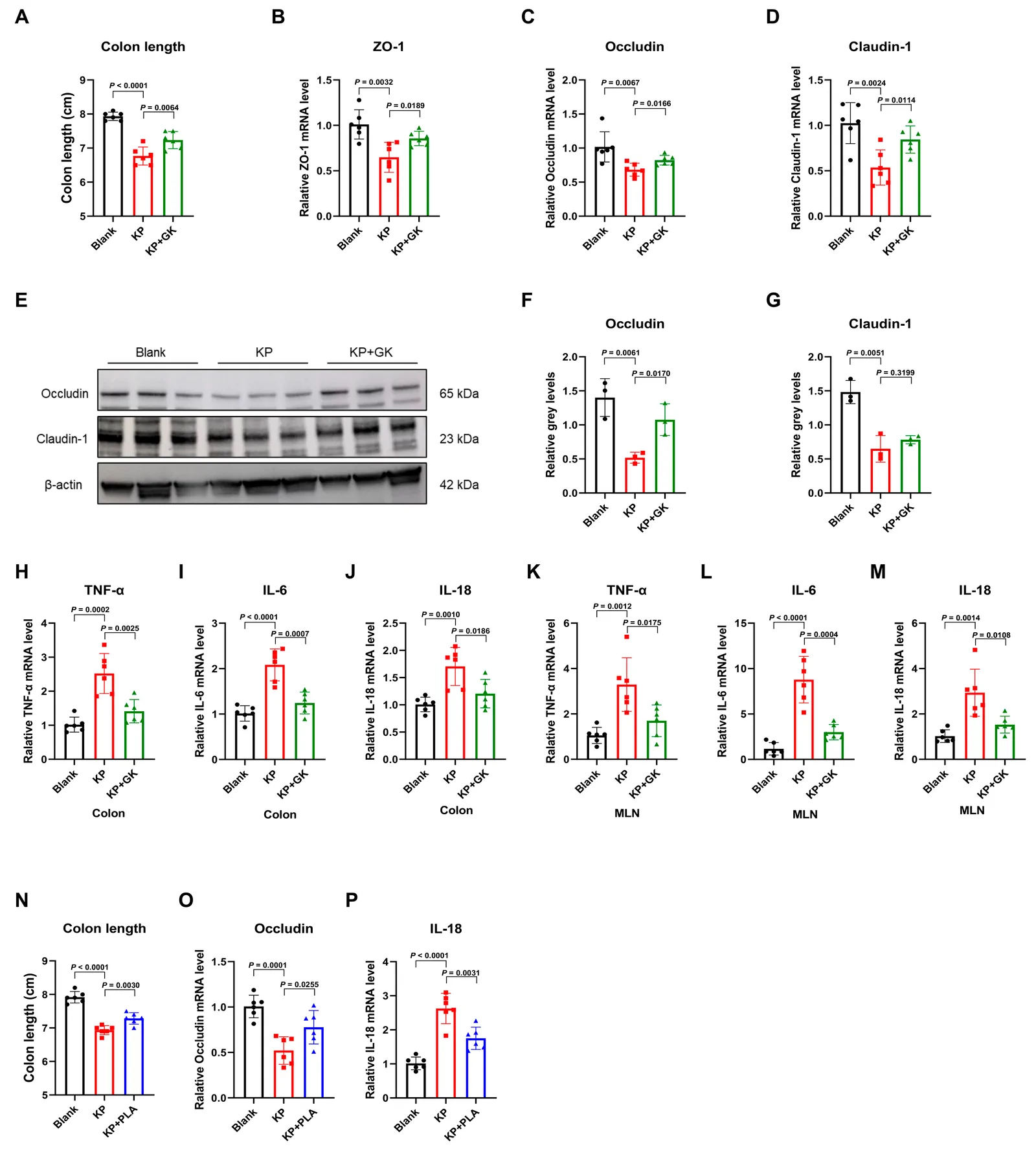
*L. plantarum* GUANKE and its metabolite PLA are associated with reduced KP-associated intestinal barrier damage and inflammation in vivo. Colon length (**A**). Relative mRNA levels of ZO-1 (**B**), Occludin (**C**), and Claudin-1 (**D**) in colon tissues. Representative immunoblot images of Occludin, Claudin-1, and β-actin in colon tissues (**E**), and corresponding quantification of Occludin (**F**) and Claudin-1 (**G**). Relative mRNA levels of TNF-α (**H**), IL-6 (**I**), and IL-18 (**J**) in colon tissues. Relative mRNA levels of TNF-α (**K**), IL-6 (**L**), and IL-18 (**M**) in MLNs. Colon length in the Blank, KP, and KP + PLA groups (**N**). Relative mRNA levels of Occludin (**O**) and IL-18 (**P**) in colon tissues from the Blank, KP, and KP + PLA groups. For colon length and RT-qPCR analyses, *n* = 6 mice per group; for Western blotting, *n* = 3 independently selected mice per group. Data are presented as means ± SD.

To evaluate the effect of *L. plantarum* GUANKE on KP-associated intestinal injury, colonic barrier function and inflammatory responses in vivo. KP challenge was associated with shortened colon length and reduced the expression of the tight junction-related molecules ZO-1, Occludin, and Claudin-1, whereas *L. plantarum* GUANKE treatment partially reversed these changes at both the mRNA and protein levels (Figure 4A–G). In parallel, KP challenge was associated with increased expression of proinflammatory cytokines in the colon and mesenteric lymph nodes (MLNs), including TNF-α, IL-6, and IL-18, all of which were attenuated by *L. plantarum* GUANKE treatment (Figure 4H–M). Moreover, exogenous PLA partially recapitulated the protective effects of *L. plantarum* GUANKE by restoring colon length, increasing Occludin expression, and reducing IL-18 expression in KP-challenged mice (Figure 4N–P). Together, these results demonstrate that *L. plantarum* GUANKE is associated with reduced KP-associated intestinal barrier disruption and inflammation in vivo, with exogenous PLA partially reproducing these effects, supporting a contributory role for PLA.

### 3.4. Influenza Enhances Host Susceptibility to Intestinal KP Challenge, Which in Turn Exacerbates Influenza-Associated Pneumonia

**Figure 5:**
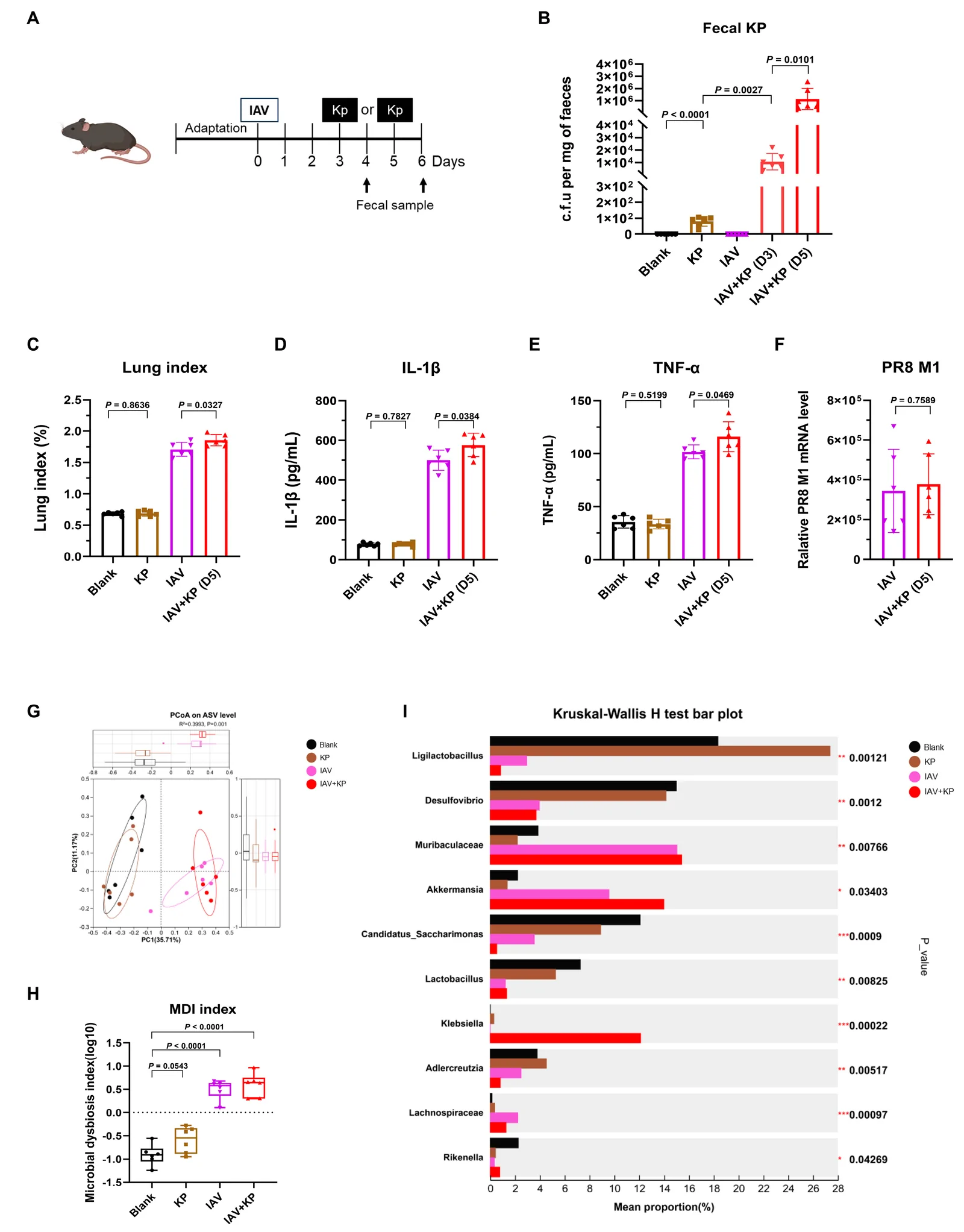
Influenza enhances susceptibility to intestinal KP challenge, and KP challenge aggravates influenza-associated pulmonary inflammation and gut microbiota dysbiosis. Experimental design (**A**). Fecal KP burden (**B**). Lung index (**C**). Concentrations of IL-1β (**D**) and TNF-α (**E**). Relative mRNA level of PR8 M1 (**F**). PCoA (**G**) and MDI (**H**) in each group. Differential taxonomic analysis showing the relative abundance of representative bacterial taxa among groups (**I**). *n* = 6 mice per group. Data are presented as means ± SD. * *p* < 0.05, ** *p* < 0.01, *** *p* < 0.001.

Studies have shown that influenza infection profoundly disrupts the host gut microbiota and increases susceptibility to intestinal bacterial burden or expansion, thereby contributing to severe secondary bacterial complications in clinical settings [15,16]. To assess whether influenza increases host susceptibility to intestinal KP challenge, mice were infected with IAV and subsequently challenged with KP (Figure 5A). Quantification of fecal KP showed that, in mice without antibiotic pretreatment, intestinal KP challenge resulted in only low levels of fecal KP. In contrast, after IAV infection, fecal KP burden was markedly increased, and KP levels were higher when mice were challenged on day 5 after IAV infection than on day 3 (Figure 5B). Moreover, compared with IAV infection alone, IAV + KP co-exposure further increased the lung index and the levels of IL-1β and TNF-α, whereas PR8 M1 expression remained unchanged, suggesting that KP aggravates influenza-associated pulmonary inflammation without significantly affecting viral burden (Figure 5C–F). Consistently, lung histopathology showed more pronounced inflammatory cell infiltration and alveolar structural disruption in the IAV + KP group than in the IAV group (Figure S2A). In addition, infectious viral titers in lung tissues were not significantly different between the IAV and IAV + KP groups, further indicating that KP co-exposure aggravated pulmonary inflammation without increasing IAV replication (Figure S2B). Selective culture of extraintestinal organs and whole blood further showed that KP was not detected in the heart, liver, spleen, lung, kidney, or whole blood, suggesting that the increased intestinal KP burden in the IAV/KP co exposure model was not accompanied by detectable systemic bacterial translocation.

To further evaluate the impact of IAV and KP co-exposure on the gut microbiota, microbial community analyses were performed. PCoA showed that the Blank and KP groups clustered relatively closely, whereas the IAV and IAV + KP groups exhibited distinct microbial community structures (Figure 5G). PERMANOVA confirmed significant overall differences among the four groups (*R*^2^ = 0.3993, *p* = 0.001). Pairwise PERMANOVA with Benjamini–Hochberg correction showed no significant difference between the Blank and KP groups (adjusted *p* = 0.331), whereas significant differences were observed between the Blank and IAV groups, the Blank and IAV + KP groups, the IAV and KP groups, and the KP and IAV + KP groups (all adjusted *p* = 0.009). A smaller but significant difference was also observed between the IAV and IAV + KP groups (adjusted *p* = 0.0468). Consistently, the microbial dysbiosis index (MDI) was significantly elevated in the IAV and IAV + KP groups compared with the Blank group, whereas no significant difference was observed between the KP and Blank groups (Figure 5H). Differential taxonomic analysis further revealed pronounced alterations in multiple bacterial taxa among groups (Figure 5I).

Together, these findings indicate that IAV infection was the predominant factor associated with disruption of the gut microbial community, whereas intestinal KP challenge alone had a limited effect in mice with an intact microbiota. IAV/KP co-exposure further altered the IAV-associated microbial community and aggravated pulmonary inflammation.

### 3.5. L. plantarum GUANKE Reduces Intestinal KP Burden and Attenuates Pulmonary Inflammation in IAV/KP Co-Exposed Mice

**Figure 6:**
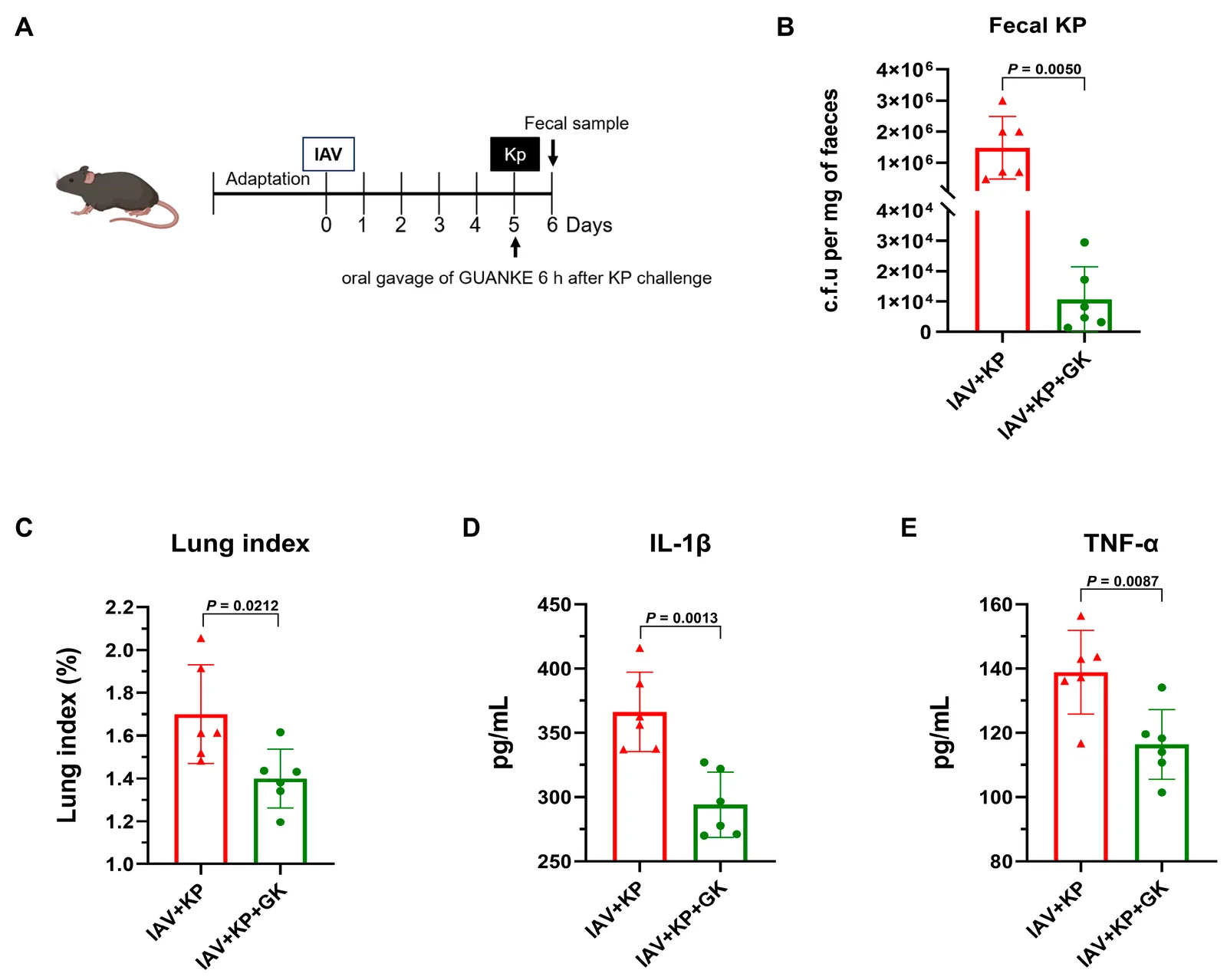
Oral administration of *L. plantarum* GUANKE reduces intestinal KP burden and is associated with lower pulmonary inflammatory markers in IAV/KP-exposed mice. Experimental design (**A**). Fecal KP burden in the IAV + KP and IAV + KP + GK groups (**B**). Lung index (**C**). Concentrations of IL-1β (**D**) and TNF-α (**E**). *n* = 6 mice per group. Data are presented as means ± SD.

Because the preceding experiment established that IAV infection increased susceptibility to intestinal KP expansion, this experiment was designed to specifically determine whether oral *L. plantarum* GUANKE could reduce intestinal KP burden under IAV/KP intestinal co-exposure conditions. Therefore, two groups of mice were included: the IAV + KP group and the IAV + KP + GK group (Figure 6A). *L. plantarum* GUANKE treatment significantly reduced fecal KP burden compared with the IAV + KP group, indicating that *L. plantarum* GUANKE reduces intestinal KP burden during IAV/KP co-exposure (Figure 6B). In parallel, the IAV + KP + GK group showed lower lung index and lower concentrations of IL-1β and TNF-α than the IAV + KP group (Figure 6C–E). These results suggest that oral *L. plantarum* GUANKE reduced intestinal KP burden and was associated with lower pulmonary inflammatory markers in IAV/KP-exposed mice.

## 4. Discussion

This study investigated the effects of *L. plantarum* GUANKE and its metabolite PLA on intestinal KP burden and associated tissue injury using in vitro assays and mouse models. We found that PLA was enriched in *L. plantarum* GUANKE fermentation supernatant, while both the fermentation supernatant and exogenous PLA inhibited KP growth in vitro. In vivo, *L. plantarum* GUANKE reduced intestinal KP burden, was accompanied by changes in gut microbiota composition, increased intestinal PLA levels, and was associated with reduced KP-associated intestinal barrier damage and inflammation. PLA partially recapitulated these protective effects, suggesting that PLA is a candidate contributing metabolite that may participate in the anti-KP activity of *L. plantarum* GUANKE. Moreover, IAV infection rendered mice more susceptible to intestinal KP expansion following oral KP challenge, and IAV/KP co-exposure was associated with increased pulmonary inflammatory markers, whereas oral *L. plantarum* GUANKE reduced intestinal KP burden and was associated with lower pulmonary inflammatory markers in the IAV/KP intestinal co-exposure model.

Lactobacilli can inhibit pathogens through secreted antimicrobial metabolites and extracellular bioactive compounds [44,45,46]. Previous studies have shown that these inhibitory substances include organic acids, bacteriocins and other proteinaceous antimicrobial substances, hydrogen peroxide, exopolysaccharides, biosurfactants, and secreted proteins, which may suppress pathogen growth by lowering environmental pH, disrupting bacterial membranes, inducing oxidative stress, or interfering with adhesion and biofilm formation [47,48,49]. In the present study, the inhibitory activity of *L. plantarum* GUANKE against KP was associated with acidity. Cell-free fermentation supernatant strongly inhibited KP growth, and heat treatment did not abolish this activity, suggesting the involvement of heat stable extracellular factors. pH adjustment experiments showed that neutralization of the *L. plantarum* GUANKE supernatant substantially weakened its antibacterial effect, indicating that acidity contributed importantly to KP inhibition. Metabolomic analysis showed that PLA was enriched in *L. plantarum* GUANKE fermentation supernatant and exhibited the strongest negative association with viable KP counts among the detected organic acids. However, the concentration of PLA detected in the *L. plantarum* GUANKE fermentation supernatant was approximately 0.2 mg/mL, whereas purified PLA produced marked inhibition of KP only at higher concentrations, beginning at approximately 0.7 mg/mL. Moreover, supplementation of 0.2 mg/mL purified PLA into MRS medium did not significantly affect viable KP counts. Two possible explanations may account for this quantitative discrepancy. First, commercially purified PLA added to MRS medium may not fully reproduce the form or physicochemical state of PLA naturally produced in the *L. plantarum* GUANKE fermentation supernatant. Second, the complete fermentation supernatant contains not only PLA but also other organic acids, and additional fermentation derived metabolites, which may act together to inhibit KP growth. Therefore, PLA should be regarded as one possible contributor rather than the main or sole mediator of GUANKE associated KP inhibition, while the overall antibacterial activity likely reflects the combined effects of acidity and multiple fermentation derived metabolites.

This study demonstrates that *L. plantarum* GUANKE exerts multilayered protective effects on intestinal homeostasis in vivo. *L. plantarum* GUANKE intervention not only significantly reduced the intestinal KP burden, but also improved indicators of intestinal barrier integrity, as evidenced by recovery of colon length, upregulation of tight junction-related molecules, and reduced levels of proinflammatory cytokines in the colon and MLNs. It has been reported that barrier disruption and inflammatory imbalance are key processes associated with aggravated tissue injury following intestinal pathogen challenge, whereas preservation of epithelial integrity and suppression of excessive inflammation contribute to the restoration of intestinal function [10,50,51]. Notably, exogenous PLA was able to partially recapitulate the protective effects of *L. plantarum* GUANKE, which is consistent with the reported roles of microbe-derived metabolites in suppressing pathogens, maintaining barrier function, and regulating inflammatory responses [35,36,52,53,54,55].

Previous studies have shown that IAV infection can markedly increase host susceptibility to secondary bacterial infection and is often accompanied by gut microbiota dysbiosis and mucosal immune imbalance [18,56,57,58,59]. The present study further showed that IAV infection rendered mice more susceptible to intestinal KP expansion following oral challenge in the absence of antibiotic pretreatment and that KP burden was higher when bacterial challenge was performed at a later stage of IAV infection. These findings suggest that IAV-associated disruption of microbial and mucosal homeostasis may create a transient intestinal environment that is more permissive to KP expansion. The results showed that, in healthy mice without antibiotic pretreatment, oral KP challenge alone had only limited effects on the gut microbiota and pulmonary inflammatory markers. In contrast, in the context of IAV infection, the intestinal KP burden was markedly increased and was accompanied by higher pulmonary inflammatory markers and pronounced gut microbiota dysbiosis. These findings suggest that intestinal KP exposure may contribute to pulmonary inflammatory responses in the context of IAV infection [16,22,60,61,62].

These findings identify *L. plantarum* GUANKE and PLA as promising microbiota derived interventions against intestinal KP challenge. Further studies are needed to validate these findings in clinical settings and to define the molecular mechanisms underlying their protective effects. However, this study has several limitations. First, only one multidrug-resistant KP strain, NK04152, was used, and future studies should evaluate additional KP isolates with diverse genetic and resistance backgrounds. Second, PLA depletion and rescue experiments were not performed; therefore, PLA should be interpreted as a contributing metabolite rather than the main or sole mediator of *L. plantarum* GUANKE activity. This issue will be further investigated in future studies.

## 5. Conclusions

In conclusion, *L. plantarum* GUANKE and PLA reduced intestinal KP burden and alleviated intestinal barrier disruption and inflammation in mouse models challenged with the KP strain NK04152.

## Figures and Tables

**Table 1:** Primer sequences.

Genes	Primer Sequences (5′–3′)
β-actin-F	ATATCGCTGCGCTGGTCG
β-actin-R	TTCCCACCATCACACCCTGG
PR8 M1-F	AAGACCAATCCTGTCACCTCTGA
PR8 M1-R	CAAAGCGTCTACGCTGCAGTCC
ZO-1-F	GCCGCTAAGAGCACAGCAA
ZO-1-R	TCCCCACTCTGAAAATGAGGA
Occludin-F	TTGAAAGTCCACCTCCTTACAGA
Occludin-R	CCGGATAAAAAGAGTACGCTGG
Claudin-1-F	GGGGACAACATCGTGACCG
Claudin-1-R	AGGAGTCGAAGACTTTGCACT
TNF-α-F	CTGAACTTCGGGGTGATCGG
TNF-α-R	GGCTTGTCACTCGAATTTTGAGA
IL-6-F	CTGCAAGAGACTTCCATCCAG
IL-6-R	AGTGGTATAGACAGGTCTGTTGG
IL-18-F	GACTCTTGCGTCAACTTCAAGG
IL-18-R	CAGGCTGTCTTTTGTCAACGA

## Data Availability

The data presented in this study are openly available in the National Genomics Data Center (NGDC) under BioProject accession number PRJCA060057.

## Supplementary Materials

The following supporting information can be downloaded at: https://www.mdpi.com/article/10.3390/microorganisms14091921/s1, Figure S1: Untargeted metabolomic analysis of *L. plantarum* GUANKE fermentation supernatant. PCA score plot showing the overall metabolic differences between MRS medium and *L. plantarum* GUANKE fermentation supernatant (A). Volcano plot of differential metabolites between MRS medium and *L. plantarum* GUANKE fermentation supernatant, defined by FDR < 0.05 and OPLS-DA-derived VIP > 1 (B). Normalized PLA peak intensity in MRS medium and *L. plantarum* GUANKE fermentation supernatant (C). Data are presented as means ± SD. Figure S2: IAV/KP co-exposure aggravates pulmonary pathological injury without increasing lung viral titers. Representative H&E-stained lung sections from the Blank, KP, IAV, and IAV+KP groups at 20× and 40× magnification (A). Infectious viral titers in lung tissues from the IAV and IAV+KP-D5 groups, measured as log_10_ TCID_50_/100 μL (B). Data are presented as means ± SD (n = 6). Statistical significance was set at *p* < 0.05.

## Abbreviations

The following abbreviations are used in this manuscript:

KP	*Klebsiella pneumoniae*
IAV	Influenza A virus
PLA	Phenyllactic acid
MLNs	mesenteric lymph nodes
MRS	De Man–Rogosa–Sharpe
BHI	Brain heart infusion
