# Supplementary material for: Lactiplantibacillus plantarum GUANKE and Its Metabolite Phenyllactic Acid Reduce Intestinal Klebsiella pneumoniae Dysbacteriosis and Related Gut Inflammatory Injury
Source: Microorganisms. 2026 Aug 31;14(9):1921. doi: 10.3390/microorganisms14091921 (PMC13609566; doi:10.3390/microorganisms14091921)
Supplement: Supplementary file 1 [file microorganisms-14-01921-s001.zip › microorganisms-4497001-supplementary.pdf]

Supplementary materials

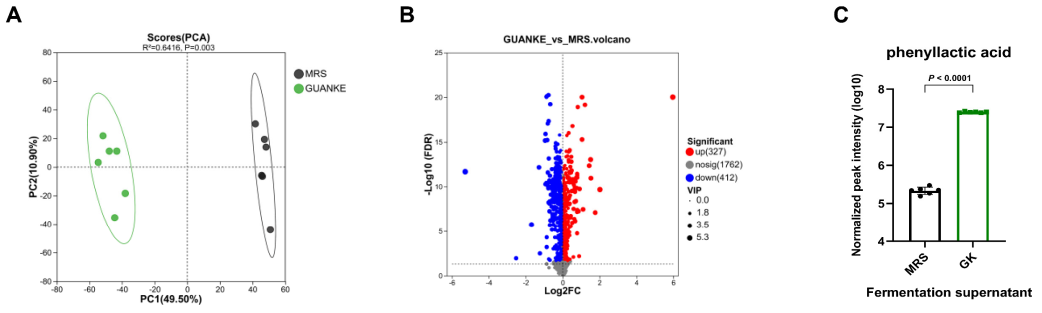

Figure S1. Untargeted metabolomic analysis of *L. plantarum* GUANKE fermentation supernatant. PCA score plot showing the overall metabolic differences between MRS medium and *L. plantarum* GUANKE fermentation supernatant (A). Volcano plot of differential metabolites between MRS medium and *L. plantarum* GUANKE fermentation supernatant, defined by FDR < 0.05 and OPLS-DA-derived VIP > 1 (B). Normalized PLA peak intensity in MRS medium and *L. plantarum* GUANKE fermentation supernatant (C). Data are presented as means ± SD.

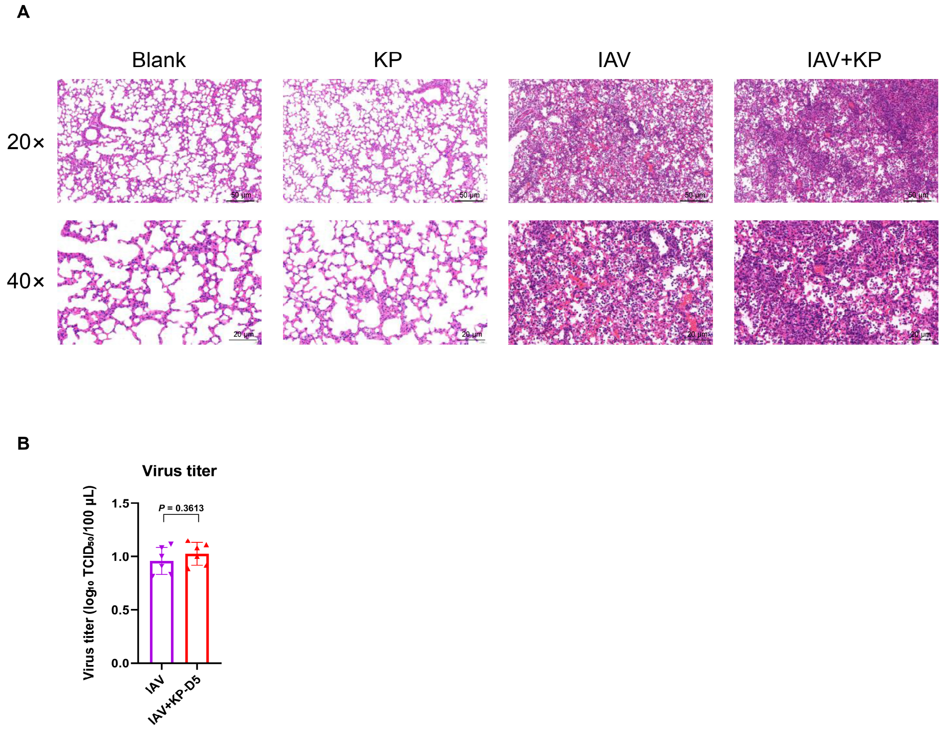

Figure S2. IAV/KP co-exposure aggravates pulmonary pathological injury without increasing lung viral titers. Representative H&E-stained lung sections from the Blank, KP, IAV, and IAV+KP groups at 20× and 40× magnification (A). Infectious viral titers in lung tissues from the IAV and IAV+KP-D5 groups, measured as $\log_{10}$ $TCID_{50}$/100 μL (B). Data are presented as means ± SD (n = 6). Statistical significance was set at $P < 0.05$.
